# Supplementary material for: Use of multi-color flow cytometry for canine immune cell characterization in cancer
Source: PLoS One. 2023 Mar 30;18(3):e0279057. doi: 10.1371/journal.pone.0279057 (PMC10062640; doi:10.1371/journal.pone.0279057)
Supplement: S1 File — (PDF) [file pone.0279057.s001.pdf]

Multicolour flow cytometry protocol for dogs

Takanori Kitamura[1], Maciej Parys[1]

[1]University of Edinburgh

1 Works for me

Reserved DOI:
10.17504/protocols.io.kqdg3p45el25/v1

maciej.parys

ABSTRACT

Although immunotherapy is becoming a standard approach of human cancer treatment, only a minor fraction of patients responds to the therapy. It is therefore required to determine the sub-populations of patients who will respond to immunotherapies along with developing novel strategies to improve efficacy of anti-tumor immune reactions. Development of novel immunotherapies is currently heavily relying on mouse models of cancer. These models are important for better understanding of mechanisms behind tumor immune escape and investigation of novel strategies to overcome it. Nevertheless, the murine models do not necessarily represent the complexity of spontaneously occurring cancers in humans. Dogs spontaneously develop a wide range of cancer types with an intact immune system under similar environment and exposure to humans, which can serve as translational models in cancer immunotherapy research. To date though, there is still relatively limited amount of information regarding immune cell profiles in canine cancers. One possible reason could be the lack of established method to isolate and simultaneously detect different immune cell types in neoplastic tissues. Here we describe a protocol for multi-color flow cytometry to distinguish immune cell types in blood, lymph nodes, and neoplastic tissues from dogs with cancer. Our results demonstrate that a 9-color flow cytometry panel enables to characterize different cell subpopulations including myeloid cells. We also show that the panel allows to detect minor/aberrant subsets within a mixed population of cells in various neoplastic samples including blood, lymph node and solid tumors. To our knowledge, this is the first simultaneous immune cell detection panel applicable for solid tumors in dogs. This multi-color flow cytometry panel has the potential to inform future basic research focusing on immune cell functions in translational canine cancer models.

PROTOCOL INFO

Takanori Kitamura, Maciej Parys . Multicolour flow cytometry protocol for dogs. **protocols.io**
https://protocols.io/view/multicolour-flow-cytometry-protocol-for-dogs-b2uqqevw

FUNDERS ACKNOWLEDGEMENT

Dogs Trust

CREATED

Dec 14, 2021

LAST MODIFIED

Aug 18, 2022

PROTOCOL INTEGER ID

55920

MATERIALS TEXT

Reagents

- 10X RBC lysis buffer (Biolegend): Dilute with distilled water (1/10) to make 1X buffer.
- MACS tissue storage solution (Miltenyi)
- Tumor dissociation kit human (Miltenyi): Reconstitute enzymes according to the manufacturer's instruction and store aliquots at –20ºC.
- Dulbecco's Modified Eagles Medium (DMEM, Gibco)
- Advanced Roswell Park Memorial Institute 1640 Medium (RPMI, Gibco), supplemented with 20% of heat-inactivated Fetal Bovine Serum (FBS, Gibco)
- Staining buffer: Dissolve 2% (w/v) bovine serum albumin (BSA, SIGMA) in Ca^{2+}/Mg^{2+} free PBS (Gibco) and filter using 500 mL filtrate bottle (Stericup-GP: 0.22μm, Millipore).
- Trypan blue stain 0.4% (Invitrogen)
- Fc receptor binding inhibitor antibody (eBioscience)
- Antibodies (details are shown in **Table 1**)
- LYNX rapid APC-Cy7 antibody conjugation kit (Bio-Rad)
- Zombie aqua fixable viability kit (Biolegend)
- Compensation beads (OneComp eBeads, eBioscience)

Materials

- Sterile needles (23G, Terumo) and 5 mL syringe (Terumo)
- Blood collection tubes (e.g., Vetlab KE EDTA, Vetlab)
- Plastics: sterile serological pipettes (5 and 10 mL, Corning), pipette tips (0.2 –1,000 μL) (Gilson, Corning), Falcon tubes (15 and 50 mL, Corning), 1.5 ml Eppendorf tubes (Sarstedt), Petri dishes (Corning), Falcon 5 mL round-bottom polystyrene test tubes with cell strainer (Corning), tissue dissociation tube (gentleMACS-C, Miltenyi), 40mm cell strainers (Corning)

Equipment

- Tools for tissue dissection (e.g., scalpel or razor blade and forceps)
- Hemocytometer or an automated cell counter (e.g. Countess, ThermoFisher)
- Centrifuge (with acceleration/brake options)
- Tissue dissociator and rotator (e.g., gentleMACS dissociator and MACSmix tube rotator, Miltenyi)
- Humidified CO_2 incubator (37 ºC, 5% CO_2)
- 14 colour flow cytometer with four lasers (violet 405nm, Blue 488nm, Green 561 nm and Red 640nm), for example BD LSR Fortessa II
- Analysis software (e.g., FlowJo, Tree Star)

Antibody list

Antigen	Clone	Fluorochrome	Host*	Isotype	Target*	Source	Volume per $2.5x10^5$ cells
**CD45**	YKIX716.13	PE	R	IgG2b	D	Bio-Rad	1.25 µL (1.25 µg)
**CD5**	YKIX322.3	FITC	R	IgG2a	D	Bio-Rad	1.25 µL (1.25 µg)
**CD21**	CA2.1D6	APC	M	IgG1	D	Bio-Rad	1.25 µL (1.25 µg)
**CD4**	YKIX302.9	PE/Cy7	R	IgG2a	D	Bio-Rad	1.25 µL (1.25 µg)
**CD8**	YCATE55.9	P.Blu	R	IgG1	D	Bio-Rad	1.25 µL (1.25 µg)
**CD11b**	M1/70	PerCP/Cy5.5	R	IgG2b	M/H#	Biolegend	0.50 µL (0.1µg)
**Neutrophil**	CADO48A	Purified†	M	IgG1	D	Kingfisher Biotech	0.25 µL (0.25µg)
**CD14**	TÜK4	AF700	M	IgG2a	H#	eBioscience	1.00 µL(0.05µg)
**MHCII**	L243	BV650	M	IgG2a	H/D	Biolegend	2.50 µL (0.25 ug)
**CD3**	CA17.2A12	FITC	M	IgG1	D	Bio-Rad	2.50 µL (2.5 ug)

***R, rat; M, mouse; D, dog; H, human**

#Cross reactivity in dog is reported (Reference #15)

†Conjugated with APC/Cy7 using LYNX Rapid APC-Cy7 Antibody Conjugation Kit (Bio-Rad)

Validated antibody panel

Sample collection and processing- blood and lymph node

1 Blood: Collect 1-2 mL of peripheral blood from the vein of dogs into tubes including EDTA.
LN: Collect fine-needle aspirate, using a 23G needle from the enlarged LN and place it into EDTA tubes with RPMI 1640 supplemented with 10% FBS.
The yield and number of aspirates depend on lymph node size and the operator skills. In our hospital, the usual yield is between $5x10^6$ to $1x10^7$, and each animal has two to three lymph node aspirates performed.

* Place the tubes on ice or at 4ºC until further processing.

2 Transfer the samples to 15 mL tubes and add 5 mL of 1x RBC lysis buffer

2.1 Incubate the tubes at room temperature for 10 minutes.

3 Add 5 mL of Staining Buffer (PBS including 2% BSA).

3.1 Centrifuge the tubes at 350 xg for 5 minutes.

3.2 Discard supernatant and resuspend the pellet with 5 mL of Staining Buffer.

3.3 Centrifuge the tubes at 350 xg for 5 minutes.

3.4 Discard supernatant and resuspend the pellet with 500 mL of Staining Buffer.

4 Filter the cell suspension through 5 mL round-bottom polystyrene test tubes with cell strainer.

4.1 Take 10 uL of cell suspension, mix with 10 uL of trypan blue, and count the cell number.

4.2 Adjust the cell density to $2.5x10^6$ cells/mL by Staining Buffer.
* The tubes including cell suspension should be kept on ice or at 4ºC.

Sample collection and processing - tumour samples

5 Transfer surgically dissected tissues into tubes including 2-4 mL of cold MACS tissue storage solution (Miltenyi).
* Place the tubes on ice or at 4ºC until further processing.

5.1 Prepare enzyme mix solution (Tumor Dissociation Kit, Miltenyi) by adding the following components into tissue dissociation tubes (e.g., gentleMACS-C tube); 4.7 mL DMEM, 200 mL Enzyme-H, 20 mL Enzyme-R, and 25 mL Enzyme-A and add it to the minced tumour sample
* DMEM should not contain Fetal Bovine Serum
* Although some epitopes can be affected by the dissociation process, there was no indication that enzymes affect the epitopes targeted by antibodies used in this panel. Moreover, these targets are stable during enzymatic treatment according to the manufacturer’s data. In case of modifications to the panel, however, a list of affected epitopes provided by the manufacturer should be referred.

5.2 Dissociate the tissue using a dissociator (e.g., gentleMACS Dissociator, h_tumor_01).
Incubate the tubes at 37ºC for 30 minutes under continuous rotation.
Dissociate the tissue by a dissociator.
Incubate the tubes at 37ºC for 30 minutes under continuous rotation.
Dissociate the tissue by a dissociator.

* Tissue dissociation condition depends on tumor type, thus monitoring of the dissociation level is recommended. In many cases, a single dissociation following 30 minutes incubation may be sufficient to dissociate the tumor completely.

5.3 Resuspend the samples and filter the cell suspensions via 40mm cell strainer placed on 50mL tubes.

6 Centrifuge the tubes at 500 xg for 5 minutes.

7 Filter the cell suspension through 5 mL round-bottom polystyrene test tubes with cell strainer.

7.1 Take 10 uL of cell suspension, mix with 10 uL of trypan blue, and count the cell number.

7.2 Adjust the cell density to $2.5x10^6$ live cells/mL by Staining Buffer.
* Keep the tubes including cell suspension on ice or at 4ºC.

Sample staining

8 Aliquot 100 mL of cell suspension to 1.5 mL tubes ($2.5x10^5$ live cells/tube)
* At least two tubes (one stained one unstained) are needed.

8.1 Add 1 uL of Fc receptor binding inhibitor antibody (eBioscience) to each tube.

8.2 Incubate the cells on ice for 30 minutes

9 Add fluorochrome-conjugated antibodies to each tube.
Antibodies required for this flow cytometry panel, along with the recommended volume of use are listed in Materials section.

9.1 Incubate the cells on ice in dark for 30-60 minutes.

10 Add 0.1mL of a viability dye (Zombie Aqua, Biolegend) to each tube

10.1 Incubate the cells at room temperature for 15 minutes in dark

11 Add 1 mL of Staining Buffer

11.1 Centrifuge the tubes at 500 xg for 5 minutes.

12 Discard supernatant and resuspend the cell pellets with 400 mL of Staining Buffer

13 Filter the cell suspension through 5 mL round-bottom polystyrene test tubes with cell strainer.
* Place the tubes on ice or at 4ºC until analysis
* Prepare single stained Compensation Beads at the same time as sample staining:
i. Mix the beads by vortexing and aliquot one drop of beads into new tubes.
ii. Add 1 uL of a single antibody into each tube.
iii. Place the tubes on ice in dark for 15-30 minutes.
iv. Add 1 mL of Staining Buffer and centrifuge the tubes at 500 xg for 5 minutes
v. Discard supernatant and resuspend the beads with 400 mL of Staining Buffer.
* Appropriate compensation is crucial for multi-colour flow cytometry assays. It should be done routinely to ensure validity of the results.

Flow cytometry

14 The protocol is validated for LSR Fortessa II (BD Biosciences) which is equipped with 4 lasers and 16 detectors, i.e., 2 detectors of the 488 nm blue laser, 6 detectors of the 405 nm violet laser, 3 detectors of the 640 nm red laser, and 5 detectors of the 561 nm yellow-green laser. Voltage for fluorescence channels is set using an unstained cell sample.

- to identify also rare populations, at least 30 000 CD45+ cells need to be captured on flow cytometer

Data analysis

15 Data can be analysed using various commercially available programs. Our group usually uses FlowJo, as it is the most user friendly, but other programs, such as FCS Express, are also potentially available.
Gating strategy is shown in the figure below

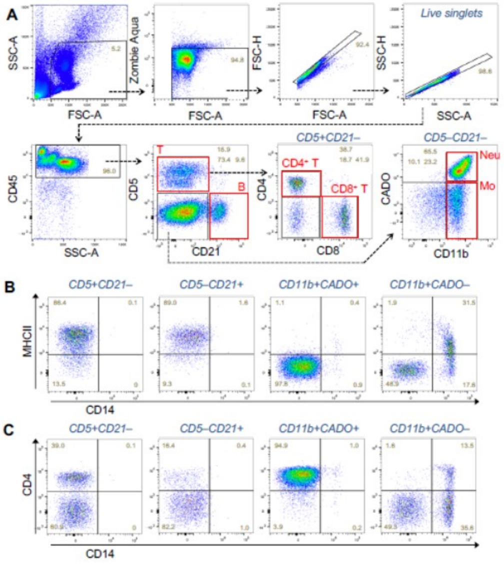

Representative dot plots showing gating strategy to detect major immune cell types in peripheral blood of dogs with peripheral lymphadenopathy. T, T cells ($CD5^{+}CD21^{-}$); B, B cells ($CD5^{-}CD21^{+}$); Neu, neutrophils ($CD5^{-}CD21^{-}CD11b^{+}CADO^{+}$); Mo, monocytes ($CD5^{-}CD21^{-}CD11b^{+}CADO^{-}$). **(B, C)** Representative dot plots showing the expression of CD14 and MHC class II (B) or CD4 (C) in each immune cell type gated in A.
