# Supplementary figures and images for: Use of multi-color flow cytometry for canine immune cell characterization in cancer
Source: PLoS One. 2023 Mar 30;18(3):e0279057. doi: 10.1371/journal.pone.0279057 (PMC10062640; doi:10.1371/journal.pone.0279057)

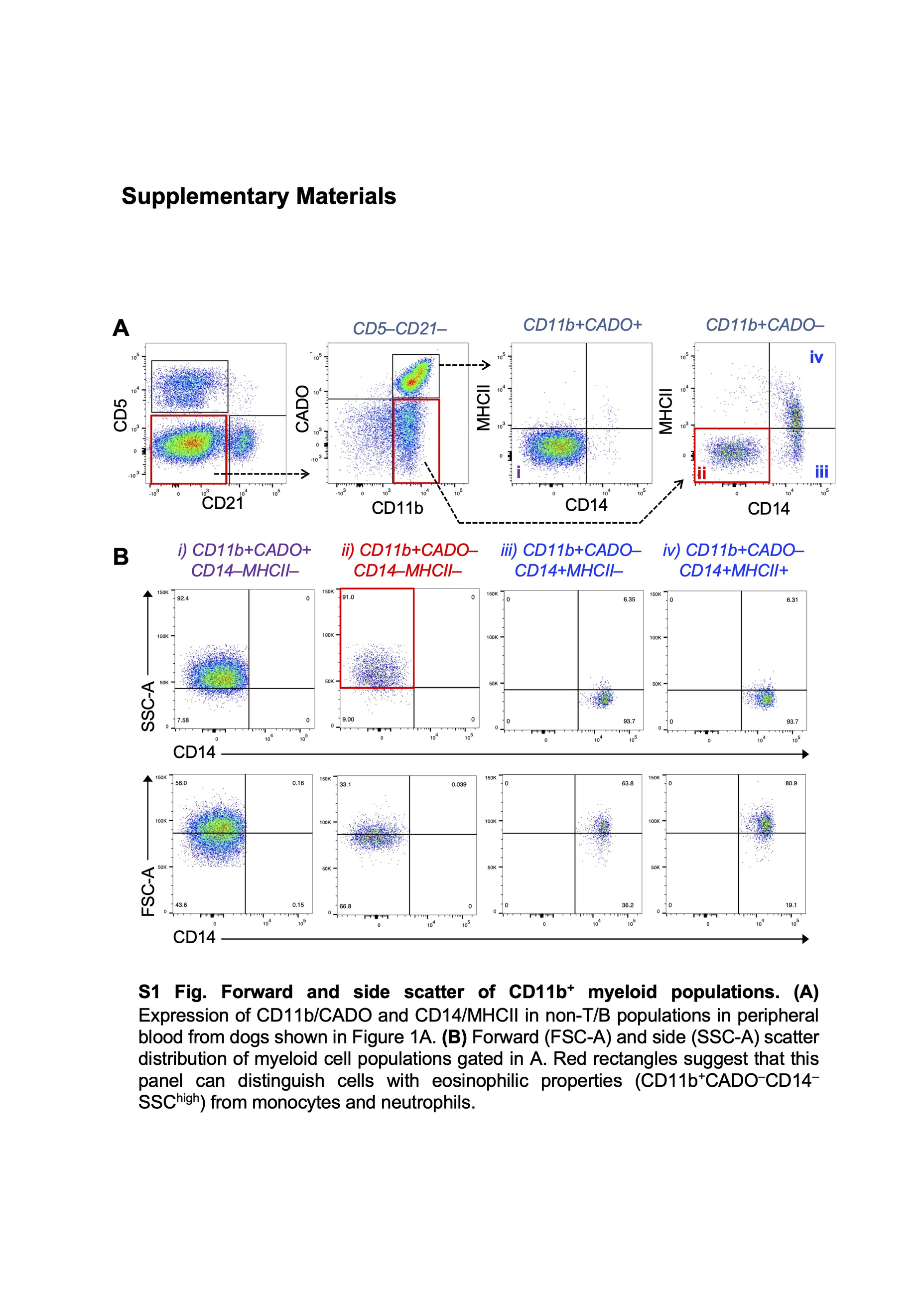

Supplement: S1 Fig — (A) Expression of CD11b/CADO and CD14/MHCII in non-T/B populations in peripheral blood from dogs shown in Fig 1A. (B) Forward (FSC-A) and side (SSC-A) scatter distribution of myeloid cell populations gated in A. Red rectangles suggest that this panel can distinguish cells with eosinophilic properties (CD11b+CADO-CD14-SSChigh) from monocytes and neutrophils. (TIF) [file pone.0279057.s002.tif]

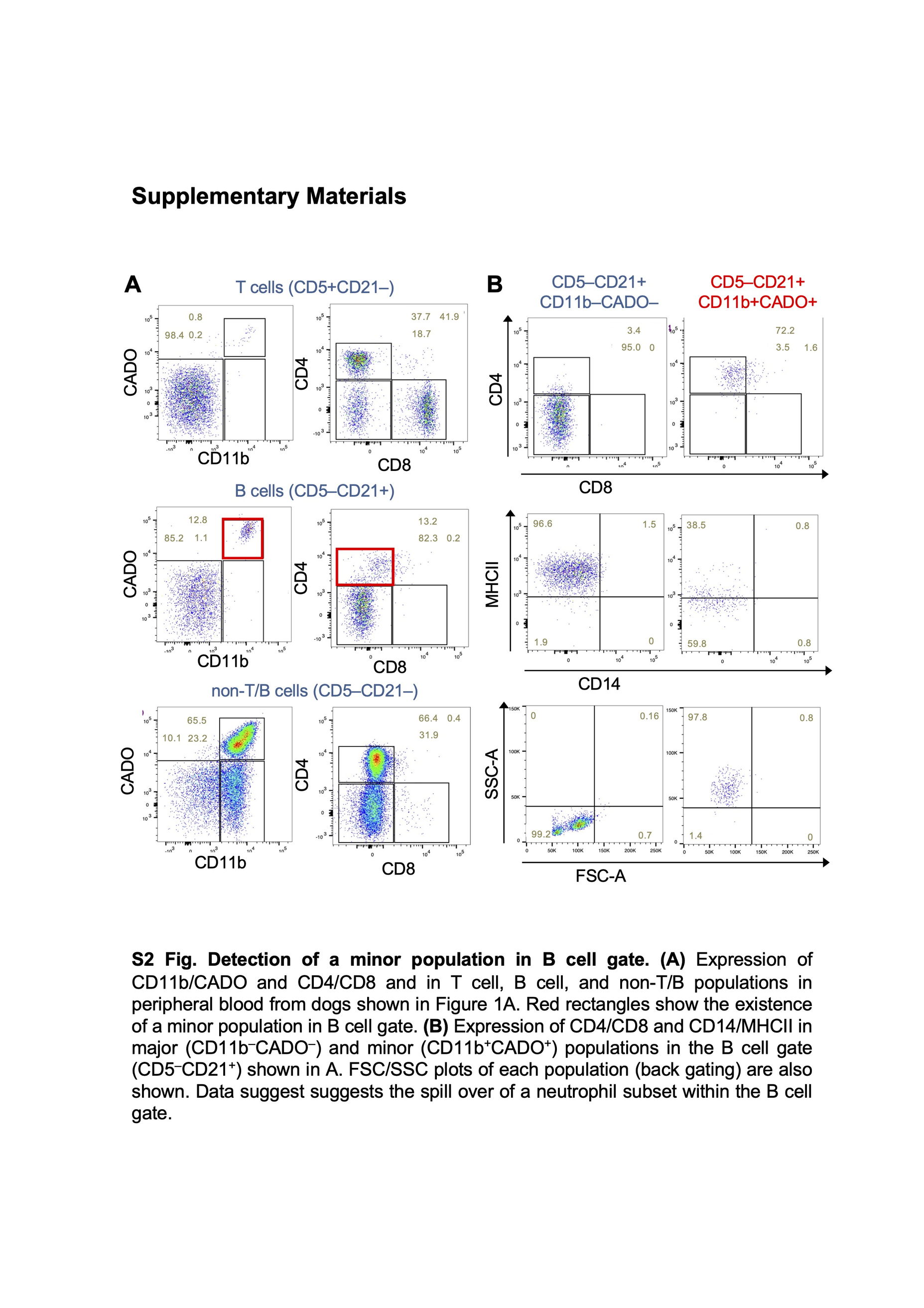

Supplement: S2 Fig — (A) Expression of CD11b/CADO and CD4/CD8 and in T cell, B cell, and non-T/B populations in peripheral blood from dogs shown in Fig 1A. Red rectangles show the existence of a minor population in B cell gate. (B) Expression of CD4/CD8 and CD14/MHCII in major (CD11b-CADO-) and minor (CD11b+CADO+) populations in the B cell gate (CD5-CD21+) shown in A. FSC/SSC plots of each population (back gating) are also shown. Data suggest suggests the spill over of a neutrophil subset within the B cell gate. (TIF) [file pone.0279057.s003.tif]

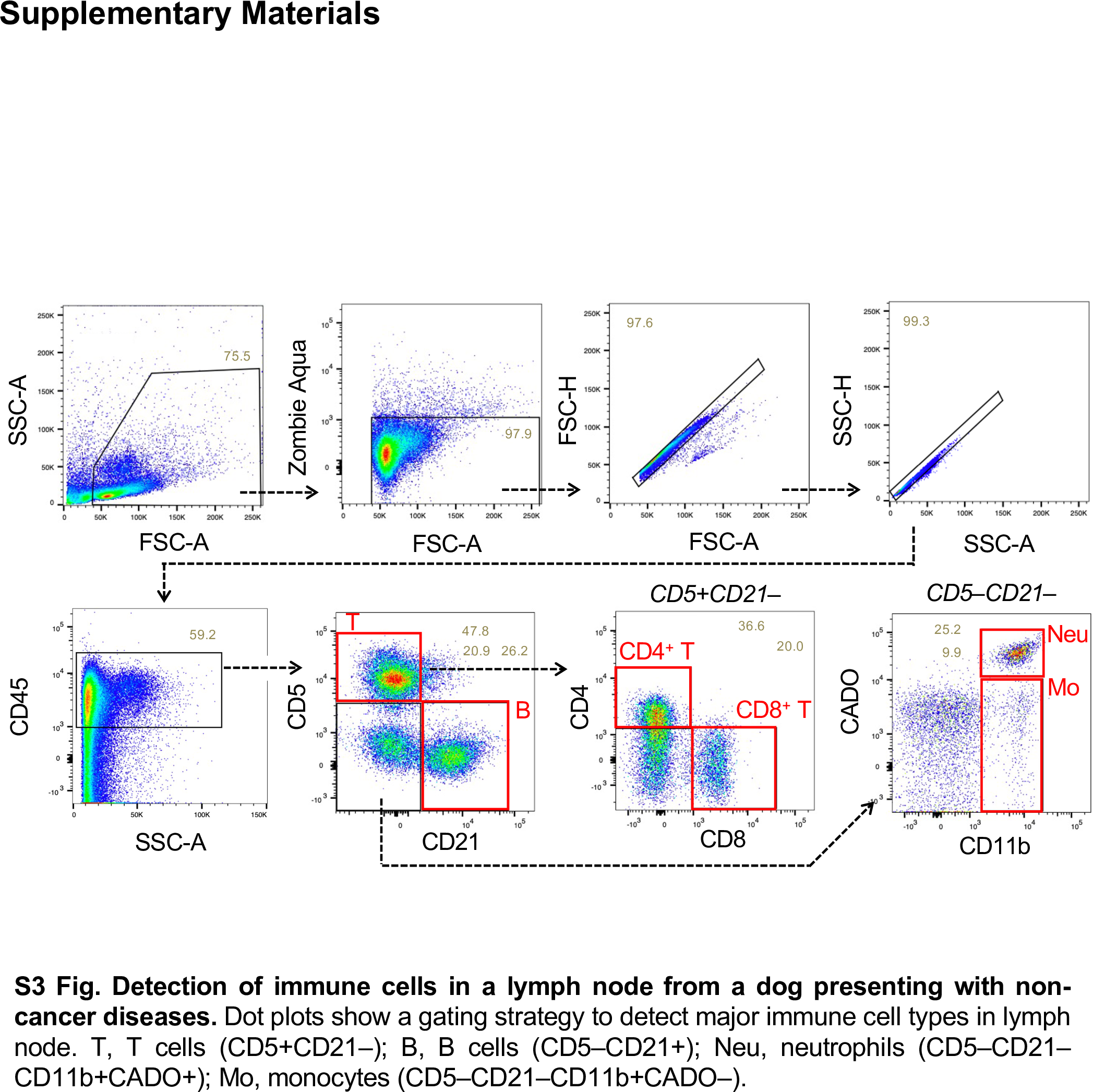

Supplement: S3 Fig — Dot plots show a gating strategy to detect major immune cell types in lymph node. T, T cells (CD5+CD21-); B, B cells (CD5 -CD21+); Neu, neutrophils (CD5-CD21- CD11b+CADO+); Mo, monocytes (CD5-CD21-CD 1 1 b+CADO-). (TIF) [file pone.0279057.s004.tif]

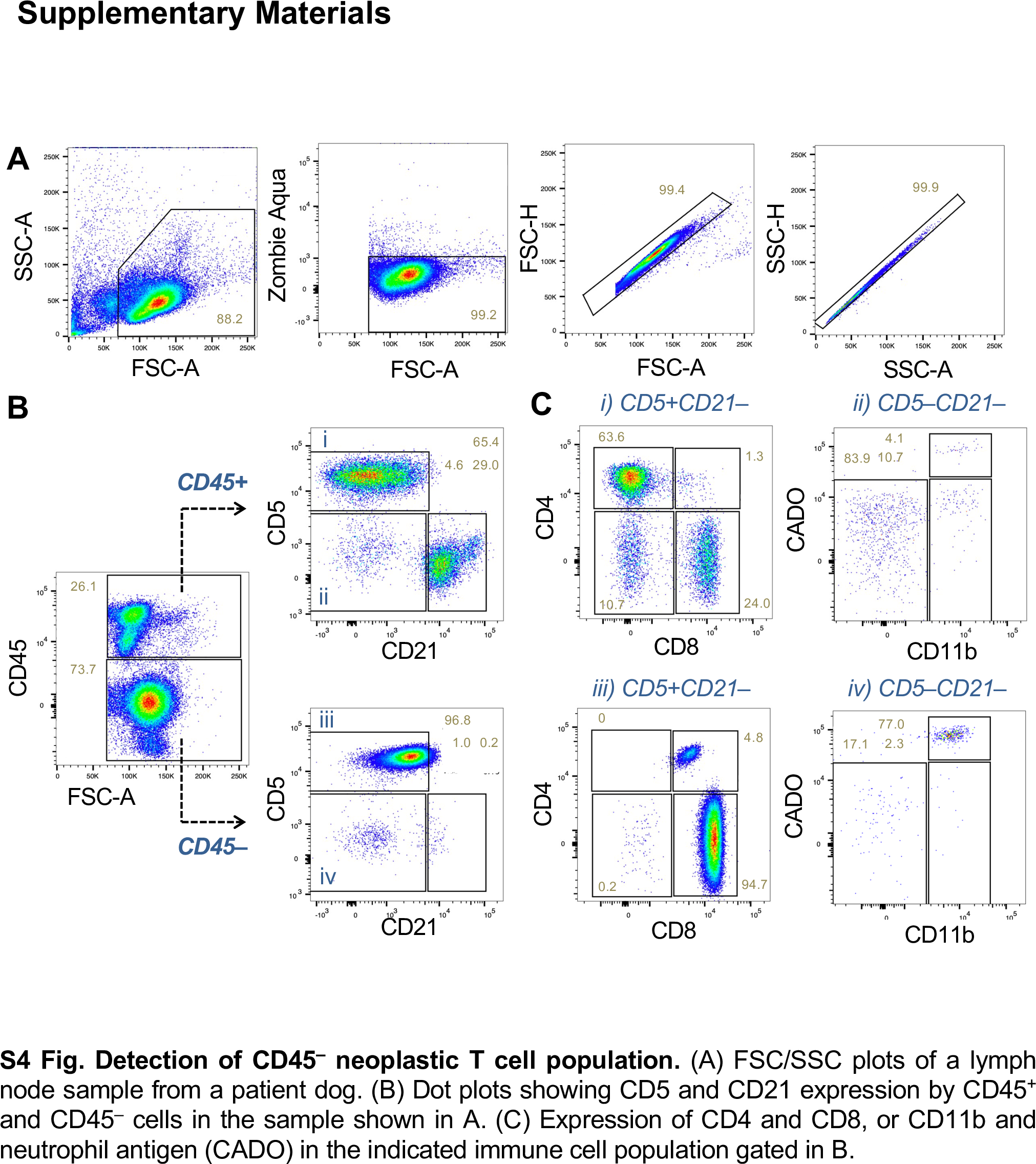

Supplement: S4 Fig — (A) FSC/SSC plots of a lymph node sample from a patient dog. (B) Dot plots showing CD5 and CD21 expression by CD45+ and CD45- cells in the sample shown in A. (C) Expression of CD4 and CD8, or CD11 b and neutrophil antigen (CADO) in the indicated immune cell population gated in B. (TIF) [file pone.0279057.s005.tif]

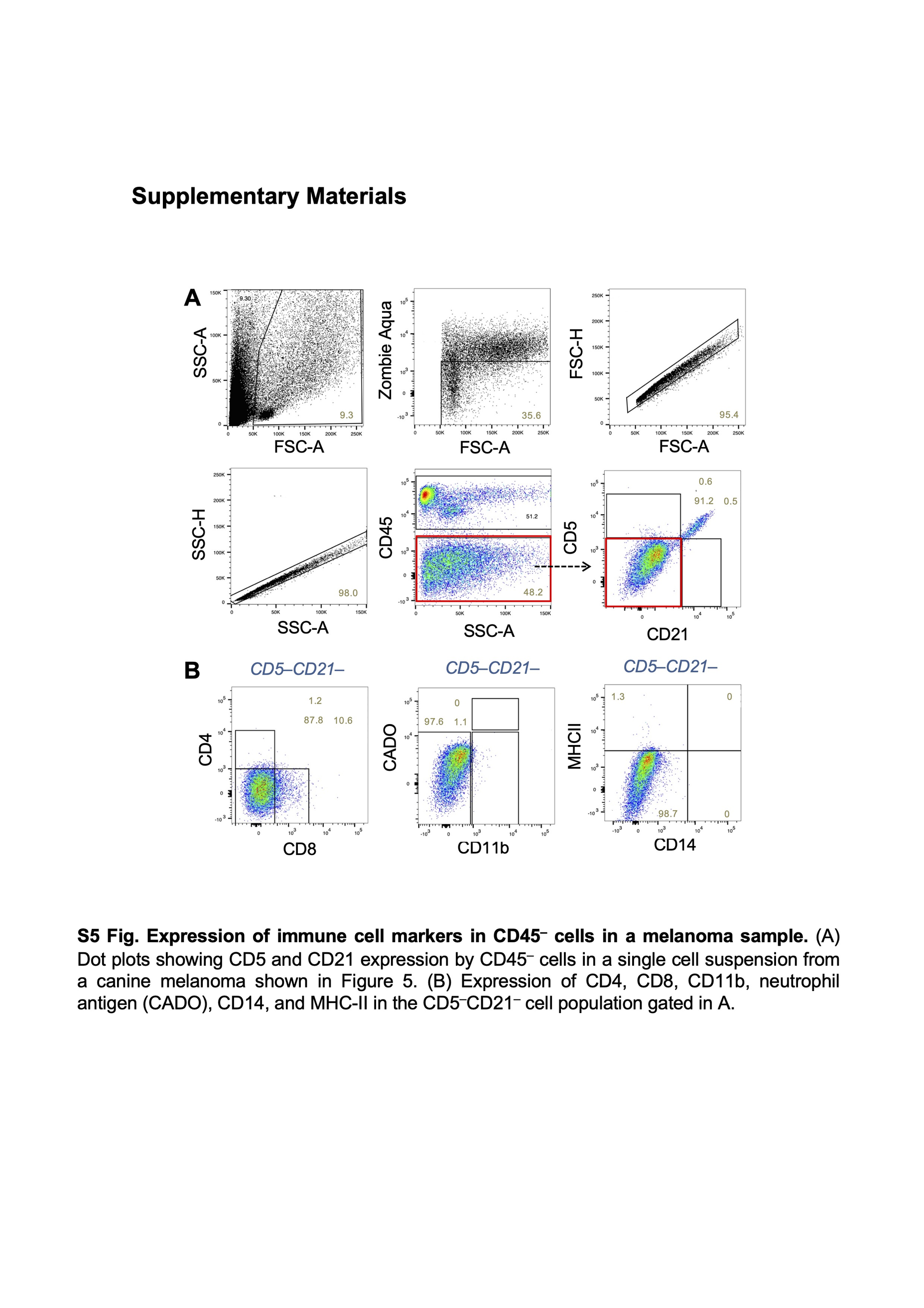

Supplement: S5 Fig — (A) Dot plots showing CD5 and CD21 expression by CD45- cells in a single cell suspension from a canine melanoma shown in Fig 5. (B) Expression of CD4, CD8, CD11b, neutrophil antigen (CADO), CD14, and MHC-II in the CD5-CD21- cell population gated in A. (TIF) [file pone.0279057.s006.tif]

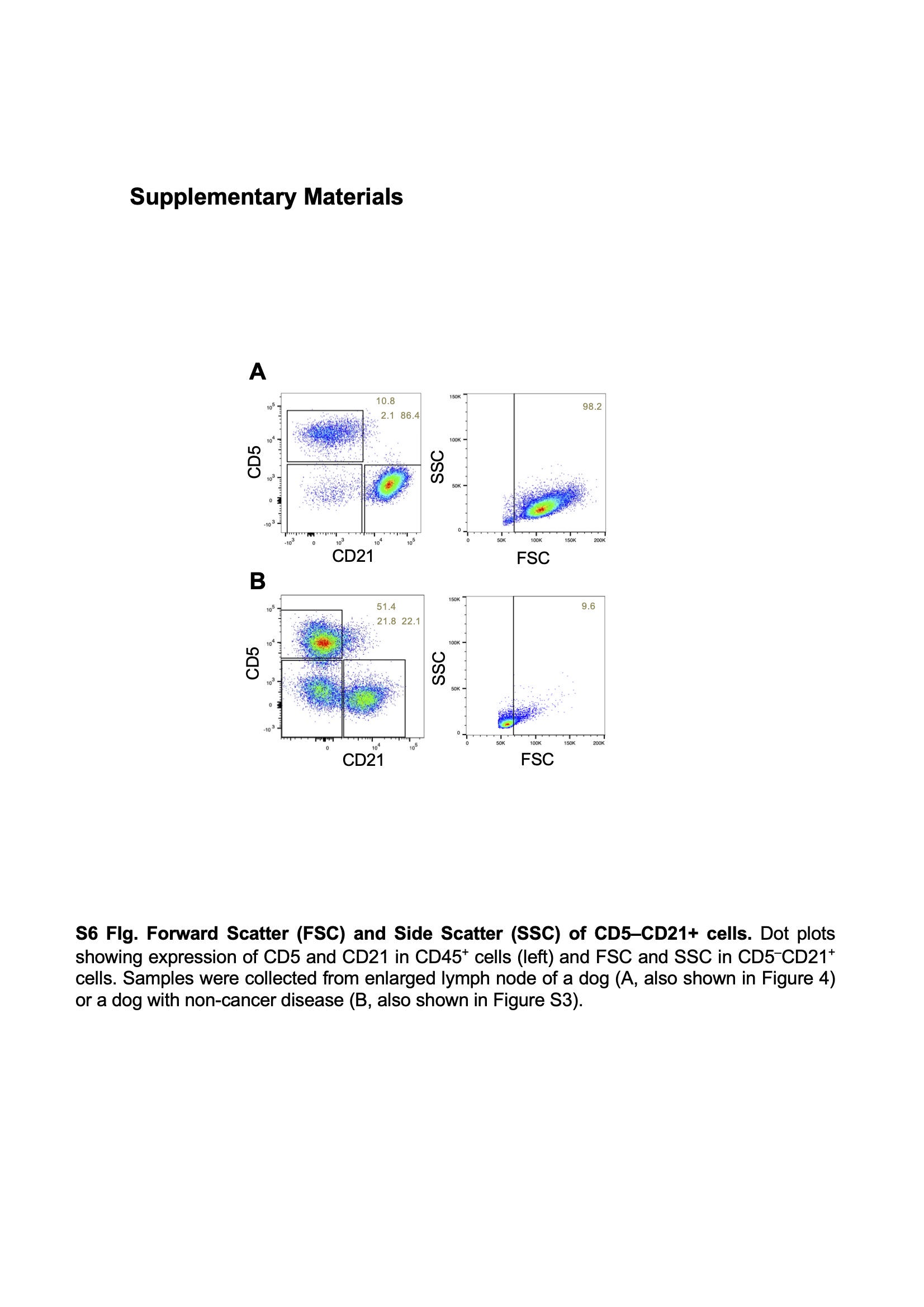

Supplement: S6 Fig — Dot plots showing expression of CD5 and CD21 in CD45+ cells (left) and FSC and SSC in CD5-CD21+. cells. Samples were collected from enlarged lymph node of a dog (A, also shown in Fig 4) or a dog with non-cancer disease (B, also shown in S3 Fig). (TIF) [file pone.0279057.s007.tif]
