# Supplementary material for: Use of multi-color flow cytometry for canine immune cell characterization in cancer
Source: PLoS One. 2023 Mar 30;18(3):e0279057. doi: 10.1371/journal.pone.0279057 (PMC10062640; doi:10.1371/journal.pone.0279057)
Supplement: Attachment — Submitted filename: Response to reviewers.doc [file pone.0279057.s008.doc]

**Responses to the reviewers’ comments**

**Reviewer #1**

This manuscript is a very straightforward methods paper describing a 9 color/marker flow cytometry panel for characterizing the major canine immune cell subsets including T and B cells, T cell subsets, and myeloid cell subsets. Furthermore, the manuscript demonstrates application of the panel to both blood and tissues, including lymph nodes and tumor lysate. The utility of the panel in canine oncology as well as other disciplines in canine medicine is without question. Methods are well described, and flow cytometry data are well presented. Details of the patient source are sparse for certain figures as mentioned below in Minor comments. Although this manuscript is not the first report describing a multicolor flow cytometry panel with more 2-4 markers for characterizing immune cell subsets in canine blood and tumor tissue in one tube as stated in the abstract, it is the first report of a panel characterizing broad categories of immune cell subsets in one tube (see comments below). Development of additional multiparameter flow cytometry panels for canine immune cells will facilitate canine cancer models that are considered highly relevant for selected human cancers. Therefore, the content of this manuscript is highly relevant to the field of translational animal models for human cancer. One weakness is the absence of data shown for a healthy control using this panel, although data is shown for canine patients with non-cancer disease. However, given the goals of the manuscript as description of new immunodiagnostic tool for canine cancer patients and illustration of the application of the tool to relevant canine disease, this weakness does not preclude the publication of the manuscript. There are minor comments listed below that should be addressed by the authors before this manuscript is accepted for publication.

🡪 Thank you very much for a positive review of our manuscript and very valuable insights. As requested, we have made the following corrections to the manuscript.

Line 3: "minor" should be modified to a "small but critical"

🡪 Requested change has been introduced into the text.

Lines 34-35; lines 41-45; lines 91-91: The statements "One possible reason could be the lack of established method to isolate and simultaneously detect different immune cell types in neoplastic tissues"; " To our knowledge, this is the first simultaneous immune cell detection panel applicable for solid tumors in dogs ," and "To date majority of studies base their insights into tumor microenvironment on immunohistochemical studies, or small, limited panels targeting single type of cells [12–14]", are somewhat arguable based on a recent publication (E. E. Sparger et al. Frontiers in Veterinary Science, December 2021, Vo. 8, Article 772932). This report describes 7 and 8 color/marker panels (one tube) to characterize T cell subsets in blood compared to T cell subsets analyzed in melanoma cell lysates from the same patients using a 5 color/marker panel for tumor. Data presented revealed differences in blood versus tumor for specific T cell subsets. Although, this recent report focused on T cell subsets (activation, regulatory and functional markers), rather than broad categories of immune cell subsets, the report should at least be cited and addressed in the Introduction. Strengths of the panel described for this manuscript in review, is the characterization of multiple immune cell subsets rather T cells only, in one tube which can be emphasized and contrasted in the Introduction and Discussion.

🡪 The sentences in Abstract (lines 34-35 and 41-45 in original manuscript) has been modified to “One possible reason could be that there are hardly any established methods to isolate and simultaneously detect a range of immune cell types in neoplastic tissues. To date only a single manuscript describes characterization of immune cells in canine tumour tissues, concentrating solely on T-cells.”.

🡪 The sentence in Introduction has been modified as following, and the suggested study has been referenced. “A recent study has described a 5-color flow cytometry panel that enables to reveal differences between T cell subsets in the blood and those in melanoma tissues [12]. However, this panel is designed to characterize specific T cell subsets, rather than broad categories of immune cell subsets.”.

🡪 Given the reviewer’s suggestion, we have also modified the last sentnce to “The approach described in this study enables the characterization of multiple immune cell subsets rather T cells only in one tube, which will help to build new insights into tumor microenvironment in translational cross-species studies.”.

Line 34: "of established" should be corrected to "of an established."

Line 39: "allows to detect" is better worded as "allows detection of."

Line 57: "barely" is better worded as "rarely."

Line 62: "environment" should be corrected to "environments."

Line 79: "distinct" should be corrected to "distinguish."

Line 87: "method" should be corrected to "methods."

🡪 Thank you for the suggestions. We have made all the requested changes in the text.

Figure 1: The data shown in Fig. 1 is identical to that shown in the S1 file where the data is stated to represent dogs showing peripheral lymphadenopathy. Accordingly, the Fig. 1 legend should also include this description. Furthermore, this description of the source for this data is too vague but is clarified by the description of the same patient suggesting a B cell lymphoma, in Figure 4 which shows data from a lymph node aspirate. Clarification of the source of data for Fig. 1 should be better described, at least within the Fig. 1 legend.

🡪 The figure legend was updated to “…Representative dot plots showing gating strategy to detect major immune cell types in peripheral blood of dogs with enlarged lymph node and diagnosed with B-cell lymphoma. …”

The MHCII- population within the CD11b+CADO-CD14+ (monocyte) population is interesting and this phenotype is suggestive of monocytic myeloid derived suppressor cells (MDSC) based on this reference (A. E. Mengos et al.; Frontiers in Immunology; May 2019; Vol. 10; Article 1147). Mention could be made in the Discussion that this population might include this immunosuppressive subset. Conversely this monocyte phenotype might also contain classical monocytes based on reference #16.

🡪 We have added the following sentence with the suggested citation. “The subpopulation of CD14^+^ monocytes defined by low expression of MHC-II might include monocytic myeloid derived suppressor cells [18].”

Lines 136-137: The reference (#16) cited in the statement, "Consistent with previous reports [16], most of neutrophils and a subset of monocytes expressed CD4 at comparable level with T cells" is not appropriate as this specific reference does not mention or test CD4 expression in canine monocytes.

🡪 We apologize for the mistake in citation. We have modified the citation to the appropriate one.

Lines 157-164: Description of raw data for immune cell frequencies in 4 dogs presenting with non-cancer-related disease, seems out of place as similar data is not presented for healthy controls or the 12 cancer patients. The goals of this manuscript are to describe a 9 color/marker panel for assessment of immune cell subsets within blood, lymph node, and tumor lysate of canine cancer patients, and to illustrate application of a such a panel to these tissue samples. However, presentation of descriptive statistics for a non-cancer cohort as shown in this paragraph, could be justified as to how the data generated by this panel could be analyzed across different patient cohorts and healthy controls. With this in mind, lines 163-164; "distinguish major immune cell types at once (etc)" could be edited to "characterize ranges for frequencies of major immune cell types across different patient cohorts with data acquired from a single panel (in one tube). This is not achievable by commonly used single- or triple-color flow cytometry."

🡪 Thank you, we have edited the text as requested.

Lines 167-177; Figure 2: Mention should be made that the data shown in Fig. 2 represents a T cell leukemia in this Results section.

🡪 The change requested have been introduced to the figures’ description.

A few additional details are warranted on sampling by lymph node aspiration for assessment by flow cytometry. Details such as cell count yields typically necessary for sufficient analysis or cell acquisition would be very helpful for the readers. Also, were multiple aspirates needed to harvest sufficient cells for staining and acquisition? Were such aspirates attempted on enlarged draining lymph nodes associated with canine patients with oral melanoma? These details were not described in the S1 file.

🡪 We have introduced changes to the protocol (S1 file) where we described the number of aspirates and how many cells are obtained on majority of aspirates.

S3 Figure: The plot for the CD5/CD21 cross-gating of CD45+ cells shows the frequencies of each population but the order of the frequencies as shown in the plot appears to be incorrect. Please clarify.

🡪 Thank you for pointing this out. We corrected the frequency of cell population in the plot.

Figure 5B. No mention is made of the CD8+ population observed within the CD5-CD21- population. Although no definitive identification is possible for this CD8+CD5- population given the markers in the panel, recent reports have shown a subset of canine NK cells to be CD8+ similar to human NK cells. This CD8+CD5- population should at least be mentioned as an observation for the lymph node of this patient and the possible identity of this population as NK cells could be mentioned as well.

🡪 Thank you for the constructive suggestion. We have added the following text and citation to the text: “Additionally, a subset of cells expressed CD8, but was negative for CD5 or CD21. Given a recent report [20], these cells may represent a subset of NK cells, which further expands the capability of the assay described here.”

S6 Figure legend. In the last line, "(B, also shown in Figure S4)," S4 should be corrected to S3.

🡪 Thank you, the figure legend for the figure S6 has been modified.

Line 282: "Enables to identify" should be edited to "allows identification of."

Line 290: "what" should be corrected to "which" and "number" to "numbers."

Line 303-304: "conjugation kit with fluorochrome" should be corrected to "conjugation kits with fluorochromes."

🡪 Thank you. We have edited the pointed words accordingly.

**Reviewer #2**

This protocol expands the current capabilities to be of use in the clinic. However the authors have dedicated the major component of the protocol to Supplementary information that would make send to be included in the body of the text.

🡪 Thank you for your insights into our manuscript. The protocol is added as supplement as it is a requirement of PLos One protocols.

There is no mention of the equipment needed to adapt this protocol in comparison with currently standard equipment in practice.

🡪 The protocol requires a 14-colour flow cytometer with four lasers (Violet 405nm, Blue 488nm, Green 561nm and Red 640nm) such as LSR Fortessa II. We have added this information as supplement (S1 file). Thus, this protocol is not applicable for a standard practice-based equipment and should be attempted at specialized laboratories.

Also from a comparative perspective, it would add value if the authors had provided a comparison of markers for each cell types between human, mouse and dogs.

🡪 The markers used in the manuscript are identical between species. We have added the following sentence in the Discussion. ”From comparative perspective, all the antibody combination and its targets have a direct counterpart in human and mice, allowing for use in translational studies.”
