# Supplementary material for: Use of multi-color flow cytometry for canine immune cell characterization in cancer
Source: PLoS One. 2023 Mar 30;18(3):e0279057. doi: 10.1371/journal.pone.0279057 (PMC10062640; doi:10.1371/journal.pone.0279057)
Supplement: Attachment — Submitted filename: Response to Reviewers.doc [file pone.0279057.s009.doc]

**Response to Editor Comments:**

Line 26 page 2: "Development of novel immunotherapies is currently heavily relying" should be edited to "Current development novel immunotherapies relies heavily."

🡪 We changed the sentence to "Current development of novel immunotherapies relies heavily."

Line 33 page 2: "a" should be inserted before "relatively limited amount."
🡪 Requested change has been introduced into the text.

Lines 39-40 page 2: "enables to characterize" should be modified to "enables characterization of."

🡪 Requested change has been introduced into the text.

Lines 89-90 page 4: "A recent study has described a 5-color flow cytometry panel that enables to reveal" should be modified to "A recent study has described a 5-color flow cytometry panel that enables identification of."

🡪 Requested change has been introduced into the text.

Line 93 page 5: "In here" should be replaced with "Herein."

🡪 Requested change has been introduced into the text.

Line 96 page 5: "a" should be inserted before "majority."

🡪 Requested change has been introduced into the text.

Line 110 page 5: "which as selected by clinician" should be modified to "which was selected by the clinician."

🡪 Requested change has been introduced into the text.

Line 269 page 12: "a" should be inserted before "general diagnostic."

🡪 Requested change has been introduced into the text.

Lines 285-286 page 13: "From comparative perspective, all the antibody combination and its targets" should be re- worded to "From a comparative perspective, all the antibodies described for this single panel along with their targets."

🡪 Requested change has been introduced into the text.

Title of the S3 figure should be edited from "in lymph node from dogs" to "in a lymph node from a dog"

🡪 Requested change has been introduced into the text.

S4B figure: the scatter plot showing initial interrogation of CD45- cells with defined populations identified as "iii" and "iv", is presumed to be gated for CD5 and CD21 as shown for the CD45+ cells. However, the axes/gates are not labelled for CD5 and CD21. This omission should be addressed

🡪 Thank you for pointing this out. We added the label in the plot.
